# Deep Learning Predicts *EGFR* Mutation Status from Histology Images in Non–Small Cell Lung Cancer

**DOI:** 10.1158/2767-9764.CRC-25-0155

**Published:** 2025-12-08

**Authors:** Jongchan Park, Sangwon Shin, Woochan Hwang, Seongho Keum, Biagio Brattoli, Jack H. Rawson, Taebum Lee, Sérgio Pereira, Chang Ho Ahn, Michael J.T. Senior, Talha Qaiser, Seokhwi Kim, Hyojin Kim, Jin-Haeng Chung, Yoon-La Choi, Se-Hoon Lee, Huw Bannister, Elia Riboni-Verri, Chan-Young Ock, Ross J. Hill, Siraj Ali, Luiza Moore

**Affiliations:** 1 Lunit, Seoul, Republic of Korea.; 2 Astrazeneca, Cambridge, United Kingdom.; 3 Department of Pathology, Ajou University School of Medicine, Suwon, Republic of Korea.; 4 Department of Pathology, Seoul National University Bundang Hospital, Seongnam, Republic of Korea.; 5 Department of Pathology and Translational Genomics, Samsung Medical Center, Sungkyunkwan University School of Medicine, Seoul, Republic of Korea.; 6 Division of Hematology-Oncology, Department of Medicine, Samsung Medical Center, Sungkyunkwan University School of Medicine, Seoul, Republic of Korea.

## Abstract

*EGFR* mutation screening in non–small cell lung cancer (NSCLC) remains variable globally and represents a significant care gap despite international recommendations and molecular testing guidelines. Recently, the use of deep learning (DL) methods to extract clinically actionable features from routine histology images has gained regulatory approval for multiple clinical applications. Therefore, the integration of predictive DL to complement molecular *EGFR* mutation screening may improve biomarker testing rates in NSCLC. To address this unmet need, we developed and validated Lunit SCOPE Genotype Predictor, a DL model trained and tuned using more than 12,000 whole-slide images, that predicts *EGFR* mutation status from routine hematoxylin and eosin images. Using a diverse dataset (*n* = 1,461) that captures histologic subtypes, multiple whole-slide scanners, different scan magnifications, and specimen types, we report an overall area under the ROC curve (AUROC) of 0.905. The model demonstrates robust performance across specimen types (biopsies and surgical resections, 0.804 and 0.912, respectively), histologic subtypes (adenocarcinoma and non-adenocarcinoma, 0.880 and 0.801, respectively), and *EGFR* mutation subtypes (AUROC, 0.854–0.931). Additionally, across a second independent test set (*n* = 599) sourced from 11 countries utilizing five different slide scanners, Lunit SCOPE Genotype Predictor achieved a robust AUROC of 0.860. Furthermore, across a multi-scanner test set (*n* = 2,261), *EGFR* mutation predictions were concordant in 90.4% of cases among five of six frequently used slide scanners. This validation across diverse clinical settings represents a vital step toward the application of artificial intelligence–based digital pathology tools in routine clinical practice to augment molecular *EGFR* mutation screening.

**Significance:**

DL predicts EGFR mutations in NSCLC from routine histology images, achieving an overall AUROC of 0.905 and 0.860 in two independent test sets across histologic subtypes, mutation subtypes, and imaging platforms.

## Introduction

Lung cancer is the most frequently diagnosed cancer globally and the leading cause of cancer-related deaths, accounting for 18.7% of all cancer-associated deaths in 2022 (1). Non–small cell lung cancer (NSCLC) accounts for 80% to 90% of all lung cancer cases and comprises two major histologic subtypes, adenocarcinomas and squamous cell carcinomas, each associated with distinct patterns of oncogenic driver mutations (2). Oncogenic *EGFR* mutations are highly enriched in lung adenocarcinomas (LUAD), occurring at a frequency of approximately 11% to 15% and 47% to 51% across European and Asian demographics, respectively (2–5).

The seminal discovery that activating mutations in *EGFR* are associated with pronounced clinical responsiveness to EGFR tyrosine kinase inhibitors (EGFR-TKI) delineated the era of precision oncology approaches for the treatment of NSCLC (6, 7). Accordingly, targeted therapies have emerged as standard-of-care first-line treatments for *EGFR*-mutant NSCLC, with significant patient benefit relative to historic treatment with chemotherapy (8, 9). Therefore, the use of genomic testing to screen patients for actionable *EGFR* alterations is recommended with the highest level of evidence in consensus guidelines for the management of advanced NSCLC (10). Indeed, improvements in overall survival rates in lung cancer are partially attributed to the success of novel targeted therapies, particularly for oncogene-addicted NSCLC (11). Additionally, the reach of precision oncology has been extended to early-stage disease as osimertinib, a third-generation EGFR-TKI, is now approved for adjuvant therapy for resectable early-stage *EGFR*-mutant NSCLC.

However, the transition toward matched targeted therapies in NSCLC demands comprehensive genomic profiling of tumor material, often from scarce tissue specimens. Nevertheless, despite genomic testing recommendations, real-world *EGFR* mutation screening rates in advanced NSCLC continue to remain suboptimal globally (12–18). Variations in the use of single-gene testing, next-generation sequencing (NGS) panels, site-specific assay turnaround times, test failures due to inadequate tissue sampling, along with discrepancies in laboratory accreditation, all drive a global need to improve *EGFR* testing workflows (14, 15, 17, 19–21). Hence, there is an evident opportunity for the integration of deep learning (DL) models that can predict genomic alterations from digital images of ubiquitously available histology sections to aid the triage of samples with a high probability of harboring *EGFR* mutations toward genomic testing. Indeed, the growing number of DL-enabled medical devices with regulatory approval offers a promising outlook for the clinical integration of these technologies (22–24).

Although classical drug-sensitizing mutations account for 80% to 90% of *EGFR* mutations observed in NSCLC [L858R point mutation and exon 19 deletions (E19del)], 10% to 20% of patients with *EGFR*-mutant NSCLC present with atypical mutations, most notably exon 20 insertions (E20ins; refs. 25, 26). Patients harboring atypical *EGFR* mutations exhibit heterogeneous and reduced benefit from EGFR-TKIs. E20ins are associated with primary resistance to first- and second-generation EGFR-TKIs, resulting in poor patient outcomes (27). More recently, E20ins have been shown to be sensitizing to an altogether distinct class of targeted therapies, anti-EGFR monoclonal and bi-specific antibodies, which block ligand-induced activation and drive target degradation (8). Therefore, defining both the presence and nature of *EGFR* alterations is of paramount importance for managing patients with advanced NSCLC.

Here, we report the development of a DL model that is able to predict drug-sensitizing *EGFR* mutations from digital images of routinely available hematoxylin and eosin (H&E)–stained sections of NSCLC. Although previous reports have demonstrated the utility of DL models to predict *EGFR* mutation status from whole-slide images (WSI), they have focused solely on single-center cohort studies (28, 29), primary resection specimens (30), single-ethnicity cohorts (31, 32), and LUADs only (30, 31, 33, 34), while typically using only a single slide-scanning platform (30, 32, 35). Here, we advance the artificial intelligence methodology of previous studies through the integration of pathology-focused foundation models for feature extraction and multiple instance learning (MIL) classifiers into an overall ensemble model approach. Furthermore, using large, demographically diverse training sets, we demonstrate the ability of a DL model to achieve robust performance in *EGFR* mutation prediction in LUAD across diverse clinical settings, including multiple slide-scanning platforms. These advances represent a vital step toward the application of computational pathology tools in routine clinical practice to augment genomic *EGFR* testing rates in NSCLC.

## Materials and Methods

### Dataset curation and genomic testing

Inclusion criteria for both training and test datasets included availability of digitized WSIs of H&E-stained sections of either surgical resection specimens, biopsy specimens, or fine-needle aspiration (FNA) specimens; histologic diagnosis of NSCLC and histologic subtype assignment by board-certified pathologists; and confirmed *EGFR* mutational status as determined by NGS- or PCR-based genetic testing.

For NGS-based mutation detection, the following panels were used according to the manufacturer’s instructions: TSO500 (Illumina), NeoComprehensive (NeoGenomics), NeoTYPE (NeoGenomics), Oncomine Precision Assay (Thermo Fisher Scientific), CancerScan (Geninus), and Qirui Panel NGS (Qirui). For real-time PCR methods, PANAMutyper EGFR (Panagene), cobas EGFR Mutation Test v2 (Roche Diagnostics), and AuroraDx Multiplex PCR (AuroraDx) kits were used according to the manufacturer’s instructions. Subsequently, *EGFR* mutation subtypes were classified as “classic” EGFR-TKI–sensitizing mutations, including E19del and L858R; E20ins and T790M (acquired resistance associated); and uncommon *EGFR* mutations (referred to as “other mutations”).

### Training and tuning datasets

A total of 11,894 digitized WSIs of H&E-stained NSCLC specimens with genomically confirmed *EGFR* mutation status were utilized to develop the *EGFR* genotype prediction model, Lunit SCOPE Genotype Predictor (SCOPE GP, Lunit). To optimize model training configuration and ensemble strategy, we utilized a tuning set comprising 389 digitized WSIs with confirmed *EGFR* mutation status. This dataset comprised 220 WSIs from the Clinical Proteomic Tumor Analysis Consortium (CPTAC; ref. 36) open-source dataset and 169 WSIs from a US-based source.

### Test datasets

Evaluation of Lunit SCOPE GP was performed on three test sets sourced independently from the training or tuning dataset. Test set A (*n* = 1,461), the primary test dataset, was curated to reflect the clinical diversity of NSCLC samples, including multiple sites of origin, different slide scanners, and specimen types, as seen in clinical practice. Test set B (*n* = 599), collected and managed by AstraZeneca, has no overlap in source institution with the other test sets and works as a blinded external test set to validate robustness. Finally, test set C (*n* = 2,261) was created with the intention of validating the model’s ability to generalize across various whole-slide scanner types and magnifications. In test set C, each specimen was sequentially imaged using six different whole-slide scanners: Aperio AT2 and GT450 (Leica Biosystems), NanoZoomer S360MD (Hamamatsu Photonics), VENTANA DP 200 (Roche Diagnostics), Pannoramic 1000 (3DHistech Ltd.), and UFS B300 (Philips). Additionally, two independent WSIs were derived from Aperio GT450, each acquired at two different scan magnifications (20× and 40×). Comprehensive clinicopathologic breakdown of test set A, B, and C is provided in Table 2.

### Data processing

**Figure 1.**
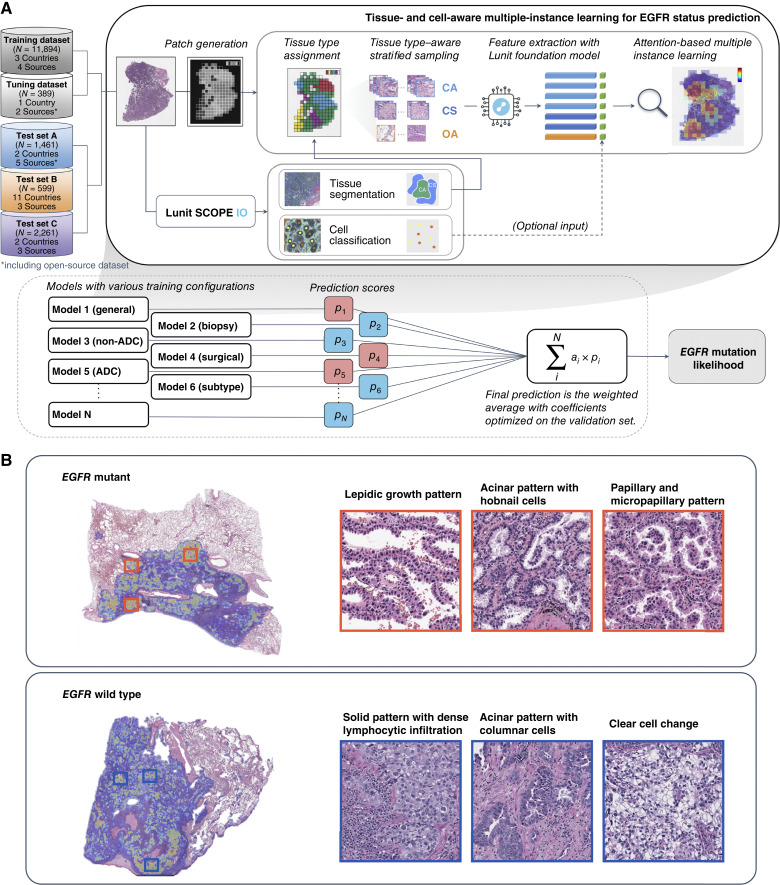
Model architecture schematic and representative whole-slide–level attention maps. **A,** Schematic of the model architecture and the ensemble strategy. WSIs of H&E-stained NSCLC samples were divided into patches. Each patch was classified as a cancer area (CA), cancer stroma (CS), or other area (OA) based on tissue segmentation results from Lunit SCOPE IO. Features were extracted using Lunit foundation models and combined with cell classification data from Lunit SCOPE IO. Feature aggregation and classification were performed using two attention-based MIL models: ABMIL model and Slot-MIL model. Predictions from multiple models trained under diverse configurations were then combined to develop the ensemble model. **B,** WSI-level attention maps with representative histologic features. Attention scores were visualized as heatmaps. For *EGFR*-mutant samples, patches with high attention scores—likely representing features strongly associated with *EGFR* mutations—are depicted in red. Similarly, patches with high attention scores in *EGFR* wild-type samples, depicted in blue, represent features associated with *EGFR* wild-type histology.

The development of Lunit SCOPE GP involves comprehensive preprocessing of WSIs through multiple sequential steps. In the preprocessing phase, WSIs undergo patch division at resolutions of either 0.5 or 0.78 microns per pixel, with square dimensions of either 224 × 224 or 1,024 × 1,024 pixels. Subsequently, the Lunit SCOPE IO model (37, 38) is used to perform two tasks. First, cell detection identifies tumor cells, lymphocytes, and other cell types. Second, tissue segmentation distinguishes between tumor areas, cancer stroma, and other tissue types, as previously validated (38). The nonoverlapping patches are then categorized by tissue segmentation results as patches primarily containing cancer area designated as CA patches, those primarily containing cancer stroma as CS patches, and the remaining patches as other patches (OA patches, normal tissue). This process is illustrated in Fig. 1A.

For feature extraction, we utilized two ConvNeXt-based (39) and one ViT-based (arXiv 2010.11929) Lunit foundation model (40): one ConvNeXt-small backbone and one ViT-L backbone are pre-trained with self-supervised methods (ONSSL-FM-CXT and ONSSL-FM-ViT) and one ConvNeXt-small backbone fine-tuned with spatial transcriptomics prediction task (ST-FM-CXT). The features from the last layer of the backbone were extracted and saved. The extracted features undergo global average pooling across spatial dimensions, yielding a one-dimensional feature vector for each patch. Following these preprocessing steps, each WSI is represented by thousands of patches, with each patch characterized by three key components: DL features, a tissue-type category, and cell detection results.

### Model architecture and training procedure

The overall architecture of Lunit SCOPE GP is illustrated in Fig. 1A. We implemented two model variants: the attention-based MIL (ABMIL) model (41) and the Slot-MIL model (arXiv 2311.17466), both utilizing attention mechanisms for feature aggregation. For model regularization and consistency with batch computation requirements in DL frameworks, we implemented a fixed-size random sampling strategy for processing input features from each WSI during the training phase. The ABMIL and Slot-MIL models employ distinct sampling methodologies to achieve this objective. The ABMIL model focuses exclusively on cancer area patches, implementing random sampling specifically among CA patch features. In contrast, the Slot-MIL model utilizes a more comprehensive stratified random sampling approach, with a predetermined distribution of 60% CA patches, 30% CS patches, and 10% OA patches. When transitioning to the inference phase, we modify this sampling approach: the ABMIL model processes all available CA patch features, whereas the Slot-MIL model incorporates all available CA patch and CS patches as inputs. The training process utilized specific hyperparameters configured for optimal model performance. Details are described in the Supplementary Materials and Methods S1.

### Ensemble strategy to improve the prediction robustness

We implemented an ensemble strategy to enhance the final model’s robustness by combining predictions from multiple models trained under diverse configurations (Fig. 1A). Each model’s training configuration incorporates three key factors: the foundation model for feature extraction (ONSSL-FM-CXT, ONSSL-FM-ViT, or ST-FM-CXT), the classifier architecture (ABMIL or Slot-MIL), and the specific subgroup used for model training (population, resection specific, biopsy specific, or non-LUAD specific). The final ensemble prediction is generated through a weighted average of individual model predictions. More details on the ensemble strategy and the performance in the tuning set are described in the Supplementary Materials and Methods S1 and Supplementary Table S1.

### Statistical analysis

The performance of the models in predicting *EGFR* mutation status was assessed using the area under the ROC curve (AUROC), along with sensitivity, specificity, positive predictive value (PPV), and negative predictive value (NPV). Ninety-five percent confidence intervals (CI) for these metrics were estimated by bootstrapping (*n* = 5,000) and are presented alongside each value. Correlations between continuous variables were analyzed using Pearson correlation coefficient (r) or Spearman rank correlation coefficient (rho). All statistical analyses were performed using R (version 4.2.3). A two-sided *P* value of < 0.05 was considered statistically significant.

### Ethics approval and consent

This study was conducted in accordance with the principles outlined in the Declaration of Helsinki and was approved by the Institutional Review Board (IRB) of Ajou University Hospital (IRB number: AJOUIRB-KS-2025-026), Samsung Medical Center (IRB number: 2022-11-094), and Seoul National University Bundang Hospital (IRB number: B-2404-893-102). All data used in this study were obtained in compliance with relevant ethical and legal standards. Data from The Cancer Genome Atlas (TCGA) and CPTAC were accessed in accordance with their respective data access policies. Data procured from commercial vendors were certified as fully de-identified in compliance with the Health Insurance Portability and Accountability Act, thus not requiring direct patient consent for this secondary analysis. Retrospective data from collaborating institutions were obtained under an IRB-approved waiver of informed consent as the research involved only de-identified data.

## Results

### Clinicopathologic features of training dataset

A total of 11,894 digitized WSIs of H&E-stained sections of NSCLC specimens with genomically confirmed *EGFR* genotypes were used as the training set for the development of Lunit SCOPE GP (Fig. 1A). Representative WSI-level attention maps are shown in Fig. 1B. Case inclusion criteria required a formal diagnosis of NSCLC and classification of histologic subtype by a board-certified pathologist as LUAD (85.4%, 10,157 of 11,894), non-LUAD [including lung squamous cell carcinoma (LSCC), 13.1%, 1,557 of 11,894], or NSCLC not otherwise specified (1.5%, 180 of 11,894). To recapitulate the variety of specimen types obtained in clinical practice, the training set included both surgical resections (44.3%, 5,269 of 11,894) and biopsies (54.8%, 6,519 of 11,894). Of the 4,524 *EGFR* mutants included in the training set, 1,682 (37.2%) had E19del, 1,734 (38.3%) had L858R point mutations, 343 (7.6%) had E20ins, 332 (7.4%) had acquired resistance–associated T790M point mutation, often in combination with other mutations, denoted “T790M,” and 161 (3.6%) had atypical *EGFR* mutations, denoted “others.” Co-mutations with variants other than T790M were identified in 271 cases (6.0%; Table 1). Importantly, the relative frequency of *EGFR* mutation subtypes present in the training dataset closely mirrors that observed in patients with NSCLC as reported by numerous genomic landscape studies (2, 42). Finally, to account for the use of different whole-slide scanning platforms in histopathology laboratories and combat modality acquisition bias, the training dataset comprised WSIs generated using five different slide scanners and included both 20× and 40× magnifications. Detailed characteristics of the training dataset are shown in Table 1.

**Table 1.** Baseline characteristics of the training set and tuning set.

Characteristic	Count (*EGFR* mutant, %)	
	Training set	Tuning set
Total	11,894 (4,524; 38.0%)	389 (109; 28.0%)
Source		
United States	3,363 (832; 24.7%)	169 (50; 29.6%)
Republic of Korea	6,531 (2,692; 41.2%)	​
China	2,000 (1,000; 50.0%)	​
Open source^a^	​	220 (59; 26.8%)
Histology		
LUAD	10,157 (4,372; 43.0%)	220 (59; 26.8%)
Non-LUAD	1,557 (87; 5.6%)	​
NSCLC-NOS	180 (65; 36.1%)	169 (50; 29.6%)
Specimen type		
Surgical resection	5,269 (2,335; 44.3%)	237 (69; 29.1%)
Biopsy	6,519 (2,132; 32.7%)	152 (40; 26.3%)
Unknown	106 (57; 53.8%)	—
*EGFR* subtype (positive counts)		
L858R	1,734	49
E19del	1,682	41
E20ins	343	3
T790M^b^	333	1
Others	161	15
L858R with other co-mutation^c^	164	—
E19del with other co-mutation^d^	98	—
E20ins with other co-mutation^e^	9	—
Magnification		
40×	10,625 (4,255; 40.0%)	142 (35; 24.6%)
20×	1,269 (269; 21.2%)	247 (74; 30.0%)
Scanner (manufacturer)		
Aperio, NOS (Leica)	819 (117; 14.3%)	389 (109; 28.0%)
Aperio GT450 (Leica)	4,080 (1,337; 32.8%)	—
Aperio AT2 (Leica)	3,019 (1,344; 44.5%)	—
UFS SG300 (Philips)	100 (41; 41.0%)	—
VENTANA DP 600 (Roche)	146 (77; 52.7%)	—
KF-PRO-120-HI (KFBIO)	2,000 (1,000; 50.0%)	—
Unknown or NA	1,730 (608; 35.1%)	—

Abbreviations: NA, not available; NOS, not otherwise specified.

a CPTAC.

b Including co-mutation with T790M.

c Co-mutation of L858R with E19del, E20ins, and other pathogenic subtypes.

d Co-mutation of E19del with E20ins and other pathogenic subtypes.

e Co-mutation of E20ins with other pathogenic subtypes.

### Curation of multicenter test sets

To validate model performance, three test sets, sourced independently from the training and tuning data, were curated to assess *EGFR* mutation status prediction. In test set A (*n* = 1,461), 969 WSIs (66.3%) were histologically classified as LUAD, whereas 492 (33.7%) were non-LUAD. A total of 1,166 WSIs (79.8%) were derived from surgical resections and 295 (20.2%) were obtained from biopsies. Of the 331 *EGFR* mutants, 150 WSIs (45.3%) had confirmed E19del, 113 (34.1%) had L858R point mutation, 21 (6.3%) had E20ins, 21 (6.3%) had T790M point mutation with or without co-mutation, and 22 (6.6%) had other mutations. Four specimens (1.2%) had co-occurring L858R mutations with either E19del or other *EGFR* mutation subtypes. The WSIs were acquired using multiple whole-slide scanners, including Aperio AT2 (*n* = 479, 32.8%), Aperio GT450 (*n* = 61, 4.2%), or Aperio, not otherwise stated (*n* = 921, 63.0%; Table 2).

**Table 2.** Baseline characteristics of the test sets (test set A, B, and C).

Characteristic	Count (*EGFR* mutant, %)		
	Test set A (*n* = 1,461)	Test set B (*n* = 599)^a^	Test set C (*n* = 2,261)^b^
Total	1,461 (331; 22.7%)	599 (229; 38.2%)	2,261 (1,176; 52.0%)
Source			
United States	211 (124; 58.8%)	190 (66; 34.7%)	1,008 (609; 60.4%)
Republic of Korea	329 (152; 46.2%)	​	1,253 (567; 45.3%)
France	​	251 (102; 40.6%)	​
Other countries^c^	​	158 (61; 38.6%)	​
Open source^d^	921 (55; 6.0%)	​	​
Histology			
LUAD	969 (318; 32.8%)	493 (209; 42.4%)	1,988 (1,127; 56.7%)
Non-LUAD	492 (13; 2.6%)	106 (20; 18.9%)	273 (49; 17.9%)
Specimen type			
Surgical resection	1,166 (208; 17.8%)	464 (155; 33.4%)	1,463 (819; 56.0%)
Biopsy	295 (123; 41.7%)	135 (74; 54.8%)	798 (357; 44.7%)
*EGFR* subtype (positive counts)			
E19del	150	82	567
L858R	113	75	378
E20ins	21	10	105
T790M^e^	21	22	63
Others	22	35	42
L858R with other co-mutation^f^	4	5	21
Magnification			
40×	1,379 (325; 23.6%)	596 (229; 38.4%)	1,938 (1,008; 52.0%)
20×	82 (6; 7.3%)	3 (0; 0.0%)	323 (168; 52.0%)
Scanner (magnification, manufacturer)			
Aperio AT2 (40×, Leica)	479 (238; 49.7%)	201 (78; 38.8%)	323 (168; 52.0%)
Aperio AT Turbo (20×, Leica)	​	3 (0; 0.0%)	​
Aperio GT450 (40×, Leica)	61 (38; 62.3%)	144 (49; 34.0%)	323 (168; 52.0%)
Aperio NOS (40×, Leica)	921 (55; 6.0%)	—	—
Aperio GT450 (20×, Leica)	—	—	323 (168; 52.0%)
NanoZoomer S360MD (40×, HAMAMATSU)	—	—	323 (168; 52.0%)
Pannoramic 1000 (40×, 3DHistech)	—	—	323 (168; 52.0%)
UFS B300 (40×, Philips)	—	150 (60; 40.0%)	323 (168; 52.0%)
VENTANA DP 200 (40×, Roche)	—	101 (42; 41.6%)	323 (168; 52.0%)

Abbreviation: NOS, not otherwise specified.

a Of the 599 samples, 101 were scanned with two different scanners [UFS B300 (Philips) and VENTANA DP200 (Roche Diagnostics)].

b From test set A, a total of 323 WSIs, originally scanned using the Aperio AT2 scanner (40×, Leica Biosystems) from 323 individual glass slides, were additionally scanned under six distinct conditions. Consequently, test set C consisted of seven sets of 323 WSIs scanned under seven distinct conditions, using different scanners: P1000 (40×, 3DHistech Ltd.), Nanozoomer 360MD (40×, Hamamatsu), Aperio AT2 (40×, Leica Biosystems), GT450 (20× and 40×, Leica Biosystems), UFS B300 (40×, Philips), and VENTANA DP 200 (40×, Roche Diagnostics).

c In the “Other Countries” category, the dataset includes 82 samples from Vietnam, 31 from Ukraine, 20 from Poland, 12 from Romania, six from Bulgaria, three from Georgia, two from Russia, one from Serbia, and one from Republic of Moldova.

d TCGA Program.

e Including co-mutation with T790M.

f Co-mutation of L858R with E19del and other pathogenic subtypes.

Model performance was further validated using an externally sourced dataset, test set B (*n* = 599). Test set B comprised 493 WSIs (82.3%) of histologically confirmed LUAD and 106 WSIs (17.7%) of non-LUAD. Additionally, the dataset included 464 (77.5%) surgical resections and 135 (22.5%) biopsy samples. Of the 229 *EGFR* mutants, 82 (35.8%) exhibited E19del, 75 (32.8%) had L858R point mutation, 10 (4.4%) had E20ins, 22 (9.6%) had T790M point mutation with or without co-mutations, and 35 (15.3%) had “other” atypical *EGFR* mutations. Five samples (2.2%) had co-occurring L858R point mutations with other *EGFR* alterations. The WSIs were acquired using five different whole-slide scanners: 201 (33.6%) with Aperio AT2, 144 (24.0%) with Aperio GT450, three (0.5%) with Aperio AT Turbo, 150 (25.0%) with UFS B300, and 101 (16.9%) with VENTANA DP 200.

Finally, test set C was curated to specifically evaluate model performance across different image acquisition modalities. To this end, 323 specimens were selected from test set A for which physical glass slides were available and subsequently scanned under seven distinct conditions (Table 2). This resulted in the generation of 2,261 unique WSIs generated using six different whole-slide scanners, one of which utilized two different scan magnifications (20× and 40×). Of the 323 specimens selected, 284 (87.9%) were histologically classified as LUAD, whereas 39 (12.1%) were non-LUAD. Surgical resections accounted for 209 specimens (64.7%), and biopsies made up the remaining 114 (35.3%). *EGFR* mutants (*n* = 168/323) exhibited the following breakdown: 81 specimens (48.2%) with E19del, 54 (32.1%) with L858R point mutation, 15 (8.9%) with E20ins, nine (5.4%) with T790M point mutation with or without co-mutation, six (3.6%) with other mutations, and three (1.8%) with co-occurring L858R point mutation and either E19del or other oncogenic *EGFR* variants. Detailed characteristics of the three test sets are presented in Table 2.

### *EGFR* mutation prediction validation

**Figure 2.**
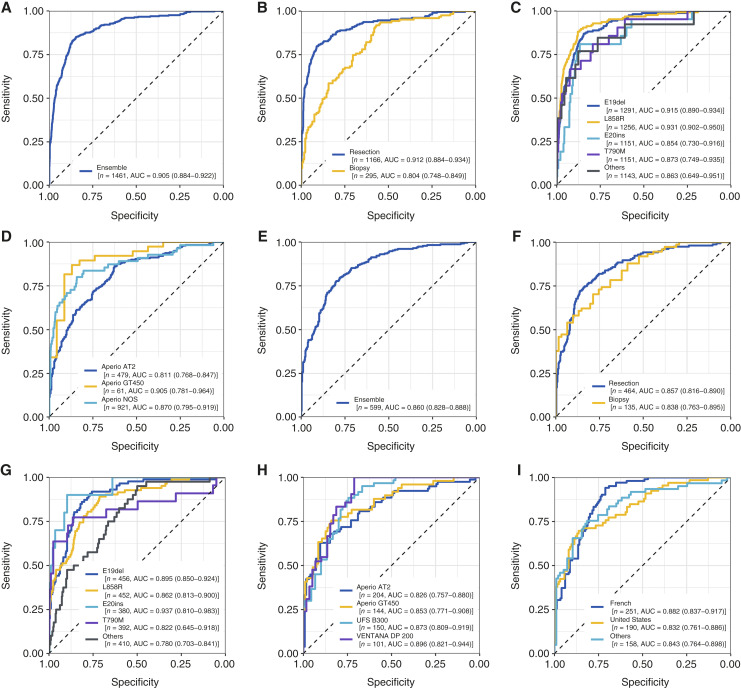
Performance of the ensemble model to predict *EGFR* mutation status across test set A (*n* = 1,461) and test set B (*n* = 599). ROC curves for test set A (**A–D**) and test set B (**E–I**) are shown. For each test set, the following subgroup analyses were performed: entire dataset (**A** and **E**), specimen type (surgical resection and biopsy; **B** and **F**), *EGFR* mutation subtypes (E19del, L858R point mutations, E20ins, T790M, and other mutations; **C** and **G**) and scanner type (**D** and **H**). For test set B, data source subgroups are additionally presented (**I**). The sample size (“*n*”) for each subgroup reflects the number of cases and controls. For *EGFR* subtypes, each ROC curve was generated using cases positive for the specific *EGFR* subtype (with or without co-mutations) as *EGFR* mutant and all *EGFR* wild-type cases as *EGFR* wild type. The data represent the AUC together with corresponding 95% CIs.

To validate the performance of Lunit SCOPE GP, we next assessed the performance of *EGFR* mutation status prediction across the three unseen test sets. Overall, the ensemble model demonstrated strong performance in predicting *EGFR* mutation status across all three test sets. In test set A (*n* = 1,461), the model achieved an overall AUROC of 0.905 (Fig. 2A; Table 3), with the distribution of prediction scores shown in Supplementary Fig. S1. The aggregate performance of the ensemble model was superior to that achieved by either of the individual sub-models (Supplementary Fig. S2). Importantly, when stratified by specimen type, the AUROC was 0.912 for surgical resections and 0.804 for biopsy specimens (Fig. 2B). Next, we stratified by *EGFR* mutation subtypes, in which the ensemble model achieved an AUROC of 0.915 for E19del, 0.931 for L858R point mutation, 0.854 for E20ins, 0.873 for resistance-associated T790M point mutations, and 0.863 for other atypical *EGFR* mutations (Fig. 2C). The distribution of prediction scores across *EGFR* mutation subtypes in test set A is shown in Supplementary Fig. S3. Next, we assessed model performance across the two most frequent *EGFR* mutation subtypes, E19del and L868R point mutation, for which we observed an AUROC of 0.925. When stratified by data source, the model achieved an AUROC of 0.801 for data from Korea, 0.882 for data from the United States, and 0.870 for data from TCGA (Supplementary Fig. S4; refs. 43, 44). Interestingly, specimens originating from Korea in test set A were more frequently biopsies, which may account for the reduced AUROC observed. Finally, we assessed model performance across the different whole-slide scanners used within test set A and observed an AUROC of 0.811 on the Aperio AT2 scanner, 0.905 on the GT450 scanner, and 0.870 on the Aperio scanner, not otherwise specified (Fig. 2D).

**Table 3.** Performance of *EGFR* genotype prediction model.

​	Test set A			Test set B		
	(*n* = 1,461)			(*n* = 599)		
	AUROC	Specificity	Sensitivity	AUROC	Specificity	Sensitivity
	(95% CI)	At sensitivity 90% (95% CI)	At specificity 90% (95% CI)	(95% CIs)	At sensitivity 90% (95% CI)	At specificity 90% (95% CI)
Total						
*EGFR* mutated	0.905 (0.884–0.922)	0.731 (0.650–0.828)	0.743 (0.671–0.815)	0.860 (0.828–0.888)	0.611 (0.510–0.699)	0.546 (0.442–0.654)
*EGFR* wild type						
Histology						
LUAD	0.880 (0.855–0.900)	0.621 (0.524–0.757)	0.632 (0.567–0.704)	0.863 (0.829–0.892)	0.592 (0.495–0.701)	0.545 (0.448–0.675)
Non-LUAD	0.801 (0.541–0.937)	0.234 (0.010–0.893)	0.692 (0.375–0.934)	0.731 (0.555–0.856)	0.140 (0.047–0.802)	0.450 (0.200–0.733)
Specimen type						
Surgical resection	0.912 (0.884–0.934)	0.708 (0.564–0.812)	0.803 (0.739–0.855)	0.857 (0.816–0.890)	0.573 (0.388–0.678)	0.568 (0.378–0.688)
Biopsy	0.804 (0.748–0.849)	0.581 (0.472–0.665)	0.423 (0.284–0.538)	0.838 (0.763–0.895)	0.525 (0.345–0.679)	0.581 (0.444–0.736)
*EGFR* mutation subtype^a^						
E19del	0.915 (0.890–0.934)	0.743 (0.675–0.858)	0.752 (0.662–0.838)	0.895 (0.850–0.924)	0.768 (0.586–0.839)	0.581 (0.410–0.740)
L858R	0.931 (0.902–0.950)	0.849 (0.684–0.891)	0.810 (0.731–0.885)	0.862 (0.813–0.900)	0.678 (0.343–0.766)	0.524 (0.407–0.641)
E20ins	0.854 (0.730–0.916)	0.600 (0.208–0.889)	0.619 (0.333–0.833)	0.937 (0.810–0.983)	0.897 (0.622–0.989)	0.800 (0.333–1.000)
T790M	0.873 (0.749–0.935)	0.651 (0.235–0.819)	0.667 (0.438–0.850)	0.822 (0.645–0.918)	0.281 (0.035–0.875)	0.636 (0.333–0.800)
Other mutations	0.863 (0.649–0.951)	0.609 (0.186–0.886)	0.615 (0.286–0.857)	0.780 (0.703–0.841)	0.524 (0.430–0.643)	0.425 (0.250–0.611)
Scanners (manufacturer)						
Aperio AT2 (Leica)^b^	0.811 (0.768–0.847)	0.506 (0.299–0.636)	0.496 (0.402–0.619)	0.826 (0.757–0.880)	0.492 (0.216–0.643)	0.628 (0.486–0.775)
Aperio GT450 (Leica)	0.905 (0.781–0.964)	0.739 (0.240–0.952)	0.816 (0.282–0.955)	0.853 (0.771–0.908)	0.495 (0.168–0.646)	0.633 (0.386–0.804)
Aperio NOS (Leica)	0.870 (0.795–0.919)	0.493 (0.244–0.808)	0.691 (0.540–0.800)	—	—	—
UFS B300 (Philips)	—	—	—	0.873 (0.809–0.919)	0.711 (0.570–0.828)	0.533 (0.358–0.787)
VENTANA DP 200 (Roche)	—	—	—	0.896 (0.821–0.944)	0.729 (0.583–0.852)	0.524 (0.281–0.773)

Abbreviation: NOS, not otherwise specified.

a The performance for a specific mutation subtype is calculated using samples with the specific mutation as positive cases and all *EGFR* wild type as negative cases (e.g., to calculate the performance of E19del, samples with only L858R cases were excluded from the analysis, rather than being included as negative cases).

b Three samples scanned using Aperio AT Turbo were included in test set B.

Comparable performance was observed across test set B (*n* = 599), for which the ensemble model achieved an overall AUROC of 0.860. When stratified by specimen type, the model achieved an AUROC of 0.857 for surgical specimen and 0.838 for biopsies. Notably, the performance for biopsy specimen exceeded that observed in test set A. Among *EGFR* mutation subtypes, the model exhibited its highest performance with E20ins, achieving an AUROC of 0.937, followed by 0.895 for E19del, 0.862 for L858R point mutation, 0.822 for resistance-associated T790M point mutations, and 0.780 for other *EGFR* alterations. When stratified by slide scanner use, the model displayed consistent performance across image acquisition platforms, achieving an AUROC of 0.826 on Aperio AT2, 0.853 on Aperio GT450, 0.873 on UFS B300, and 0.896 on VENTANA DP 200. Additionally, the model demonstrated consistent performance across diverse data sources, achieving an AUROC of 0.882 for data from France, 0.832 for data from United States, and 0.843 for data from other countries, including Vietnam and Russia (Fig. 2E–I).

### Clinical utility of *EGFR* mutation prediction

The clinical utility of our model was then assessed using PPV and NPV, with calculations performed at different *EGFR* mutation prevalence rates to reflect diverse populations (3). We conceptualized the model as a screening tool to prioritize patients for confirmatory molecular testing. Using a cutoff that achieved 90% sensitivity and 73.1% specificity in test set A (Table 3), we simulated performance based on a prevalence of 22.7%, which reflects *EGFR* mutation rates in North America and was the prevalence in test set A. Under these conditions, the model demonstrated a PPV of 49.5% (95% CI, 47.0%–52.1%) and an NPV of 96.2% (95% CI, 95.0%–97.4%). When a prevalence of 50% was assumed to simulate performance across Asian populations, the model reported a PPV of 77.1% (95% CI, 74.4%–79.7%) and an NPV of 88.0% (95% CI, 85.2%–90.8%).

### Interpretability for the *EGFR* prediction

To provide insights into the model’s interpretability, attention maps were generated by capturing the self-attention scores for all input patches. As Lunit SCOPE GP utilizes the ensemble strategy, the attention maps from each individual model were averaged with respect to the optimized ensemble weights as described in the materials and methods. Finally, the ensembled attention maps were normalized for enhanced interpretability (Fig. 1B). The attention map values can be interpreted as how much each individual patch contributed to the final *EGFR* mutation prediction score. A high attention value indicates that the model leverages more on that patch’s information. Indeed, examination of patches with high attention values from WSIs of genomically confirmed *EGFR*-mutant NSCLC cases reveals histologic features known to be associated with *EGFR* mutation, such as lepidic, papillary, and micropapillary growth patterns and hobnail cytomorphology (Fig. 1B). In contrast, examination of patches with high attention values from WSIs of *EGFR* wild-type samples reveals histologic features infrequently associated with *EGFR*-mutant NSCLC, including solid patterns with dense lymphocytic infiltration, columnar cells, and clear cell changes (Fig. 1B).

### *EGFR* mutation prediction across different whole-slide imaging conditions

**Figure 3.**
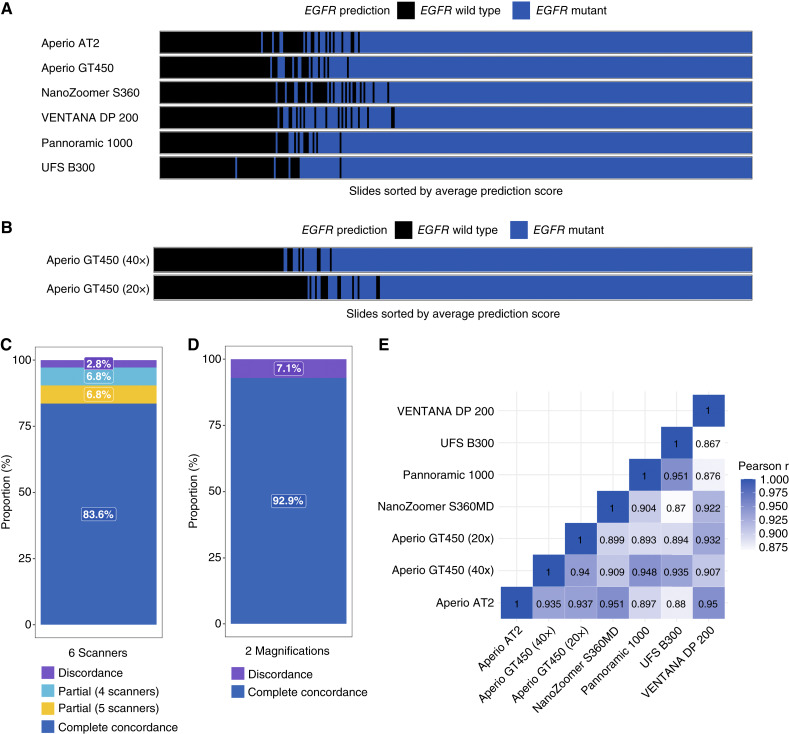
Generalized performance across different whole-slide scanners and magnifications in test set C (*n* = 2,261). **A** and **B,** Binary classification performance for *EGFR* mutation prediction across six scanner types (**A**) and two magnifications (**B**). The *x*-axis represents the slides sorted by average value of prediction scores from six scanners. The predicted *EGFR* classification determined by the model is denoted by color (blue, *EGFR* mutant; black, *EGFR* wild type). **C** and **D,** Concordance rates of binary classification performance across scanner types (**C**) and magnifications (**D**). **E,** Pearson correlation coefficients (r) for the continuous prediction scores across scanner types and magnifications.

Having demonstrated robust *EGFR* mutation prediction across different patient demographics, mutation subtypes, and specimen subtypes, we next evaluated the model’s performance across different clinical slide scanners and scan magnification. This is of particular importance as differences in scanning hardware settings and image post-processing algorithms may lead to pixel value variations and affect model performance (45). To this end, model performance was assessed using test set C (*n* = 2,261), which comprised 323 specimens sequentially imaged under seven distinct imaging conditions (six different whole-slide scanners, one of which used two different scan magnifications). Using a binary cutoff for *EGFR* mutation status, as determined by the 90% sensitivity threshold of the tuning set, we observed that *EGFR* mutation predictions from five of six scanners were consistent in 90.4% of cases, and predictions from the same scanner but acquired using two different scan magnifications were consistent in 92.9% of cases (Fig. 3A–D; Supplementary Figs. S5 and S6). Pairwise comparison of prediction scores between different slide scanners showed good overall correlation, with Pearson r ranging from 0.867 to 0.951 (Fig. 3E). The strongest correlations were observed between the Aperio AT2 and the NanoZoomer S360MD (0.951), as well as between the Pannoramic 1000 and the UFS B300 scanners (0.951). Notably, we observed a strong correlation between *EGFR* mutation predictions when using different scanning magnifications (20× and 40×) on the Aperio GT450, with r = 0.940 (*P* < 0.001). To further corroborate these data, we stratified test set B, which contained 101 specimens that were sequentially imaged using two different whole-slide scanners, the UFS B300 and the VENTANA DP 200 at 40× magnification, to assess inter-scanner performance across a second cohort. Similar to test set C, we observed a strong correlation between *EGFR* mutation predictions using two different scanners (*n* = 101) which was 91.1%, with a Pearson r of 0.905 (*P* < 0.001), with prediction agreement in 91.1% of cases. Collectively, these data demonstrate robust performance across clinically relevant whole-slide imaging modalities.

### *EGFR* mutation prediction for non-LUAD

In stark contrast to LUAD, *EGFR* mutations are observed less frequently in LSCC, with reported incidences of 0% to 5% (2, 5, 46–49). The complexity of diagnosing adenosquamous carcinoma from core needle biopsies and the misidentification of poorly differentiated LUAD as LSCC are reported to underlie the tangible *EGFR* mutation rates reported in suspected LSCC biopsies (46, 49). Consistent with these caveats, the European Society for Medical Oncology guidelines for *EGFR* testing in oncogene-addicted NSCLC specifically exclude LSCC, unless unusual clinico-pathologic factors suggest otherwise (50). Considering this, we sought to determine whether the use of DL models could predict rare *EGFR* mutations in non-LUAD specimens. To this end, we stratified test sets A and B for specimens with non-LUAD classifications, yielding 492 and 106 samples, respectively. Consistent with published reports, the frequency of *EGFR* mutations was relatively low in non-LUAD cases compared with that observed in LUAD at 2.6% in test set A and 18.9% in test set B. Interestingly, the ensemble model achieved AUROC of 0.801 and 0.731 for non-LUAD samples in test set A and B, respectively, demonstrating robust performance despite the challenging context (Table 3).

### *EGFR* mutation prediction across different tumor cell quantity

Given the robust performance achieved across diverse datasets, clinical slide scanners, and scan magnifications, we next examined the applicability of our ensemble model in samples with varying tumor cell content. This is of significant clinical importance as inadequate tumor content is a major contributing factor to genomic *EGFR* test failures (20). Therefore, deriving *EGFR* mutation predictions from otherwise limiting material is of substantial clinical benefit. To test this, test set A (*n* = 1,461) was stratified into 10 bins by deciles based on either tumor cell count or tumor cell volume as quantified by Lunit SCOPE IO. We observed an incremental increase in model performance with increasing tumor cell count, with the ensemble model achieving a bin-level AUROC of 0.800 and 0.900 at minimum tumor cell counts of 2,748 and 99,253 tumor cells, respectively (Supplementary Fig. S7). When samples were stratified based on percentage tumor volume, the ensemble model achieved a bin-level AUROC of 0.900 at minimum tumor volume of 9.85% (Supplementary Fig. S8).

Critically, these data suggest that DL models can be successfully employed to predict *EGFR* mutation status from otherwise limiting material. To further extend this observation, we stratified test set A and B to assess model performance on specimens obtained by FNA as this class of specimens exhibits reduced cellularity and a disruption of tissue architecture. To this end, we identified a total of 42 samples that were obtained via FNA, for which we achieved an AUROC of 0.732 (95% CI, 0.561–0.836). Although limited, these data demonstrate the potential utility for DL-based mutation prediction in FNA specimens.

## Discussion

For patients with advanced NSCLC, assessing the presence of drug-sensitive *EGFR* mutations is a critical step in disease management as third-generation EGFR-TKIs are the standard-of-care first-line treatment for advanced *EGFR-*mutant NSCLC. However, the use of genomic testing for sensitizing *EGFR* mutation remains suboptimal globally even in non–resource-limited settings (51). Therefore, the integration of digital pathology tools and state-of-the-art artificial intelligence models to provide rapid assessment of genomic alterations in cancer from routine histopathology sections is well poised to enhance the reach of precision oncology (52).

Here, we report the design, training, and testing of a DL model developed using, to the best of our knowledge, the largest and most clinically diverse dataset for *EGFR* mutation prediction in NSCLC to date. Trained using 11,894 gigapixel WSIs sourced from diverse clinical settings, including LUADs, non-LUADs, surgical resections, biopsies, and FNA biopsies imaged using at least five clinical whole-slide scanners and at multiple scan magnifications, we achieved an overall AUROC of 0.905 and 0.860 for two independent test cohorts (test set A and test set B, respectively). Although we include non-LUAD cases in the evaluation, the primary clinical utility of this model is within LUAD, which accounts for the vast majority of *EGFR* mutations in NSCLC. Our training data reflected this, with non-LUAD cases comprising only 13.1% of the dataset and only 5.6% of those having *EGFR* mutations.

The approach described here expands substantially on previous reports, with respect to both training and test set diversity, and the use of novel model architecture design. Specifically, through the curation of intentionally diverse training and test datasets, we aimed to facilitate the development of a robust prediction tool able to accurately predict *EGFR* mutation status across key clinical variables, including patient demographics, whole-slide imaging hardware, file formats, and pre-analytic conditions across global centers.

To strategically address the above variables, the training and tuning sets were constructed to be purposefully diverse, with specimens sourced from centers both multi- and sub-regionally, including multiple centers from the United States and for Asia, centers within China, Korea, and others. Importantly, this level of diversity was maintained throughout model testing, with test cases originating from France, China, Korea, and Vietnam. This multicenter approach also ensured demographic diversity even with nuance, which is of significant importance given the variation in *EGFR* mutation rates across global populations (53). Furthermore, previous studies have predominantly utilized large yet demographically homogenous datasets, which may lead to bias and underperformance in ethnic subgroups (54).

Analogously, we employed WSIs generated using six different whole-slide scanners to better recapitulate the variability encountered in routine clinical situations, as other studies have relied on a single scanner being used to create all or the majority of WSIs in a training set (30). This is of significant importance as DL models are prone to overfitting to domain-specific details, such as scanner-induced variations, making training reliant on a single image format likely to result in limited robustness to other scanner formats (55). Beyond the bespoke optics of image acquisition, such as sensor chips and illumination settings, scanner-specific acquisition algorithms may carry artefacts as manufacturers deploy proprietary image compression or post-processing algorithms (45). Many different scanners are used globally, with some scanners having strong regional representation (e.g., KFBIO products in China; personal communication), making representation of such nuance key. Our training set is composed of images acquired by scanners that comprise much of the global market. Although a reasonable level of concordance has been observed among clinical slide scanners and magnifications, some discordant cases remain. Future research should explore the factors contributing to inter-scanner and inter-magnification variability and focus on improving the robustness of the model across diverse imaging conditions.

Similarly, previous studies utilized performance test sets that were either sourced from the same centers/consortia used for model training or sourced independently but limited in size (28, 32). These limitations evoke the need for demographic and scanner diversity to enable translation to real-world practice. In this study, we curated three large and unseen test cohorts independently sourced by AstraZeneca and Lunit from multiple centers with both demographic and scanner diversity.

Beyond the training and test set configuration, several strategic choices in model architecture were critical for enhancing the overall performance. For assessment of features by the MIL model, we utilized an embedding-level approach to weight different instances within the bag and thus focus on the most relevant instances. This is in contrast to utilizing an instance-level approach (learning at the instance level rather than the bag level). The greater complexity of embedding is supported by operational feasibility, including computational ease and robust performance, in previous studies in an MIL model for WSI-level classification tasks (56).

We used three foundation models vigorously pre-trained on histology images with different pre-training methods (self-supervised or spatial transcriptomics prediction) and different architectures (ConvNeXt-small or ViT-L). Such foundation models, although powerful, are still an evolving field and do not yet fully demonstrate the ideal characteristics of robust few-shot or zero-shot learning and generalization across diverse settings. These models were chosen to complement each other as different architectures tend to excel at different tasks. For example, vision transformer models (e.g., ViT) are often considered effective at capturing global context and object shapes, whereas convolutional neural network models (e.g., ConvNeXt) are often considered better at capturing texture and edges (arXiv 2202.06709). The instance features extracted from the foundation models are used as MIL model inputs.

We used attention-based MIL models in which the highly attended instances contribute to the model prediction more. We used the ABMIL model given its robust performance across various tasks (56) and the Slot-MIL model to utilize information from all patches (CA, CS, or other patches). We further used an ensemble strategy to improve the robustness of the prediction scores. Empirically, we have found that ensembling prediction scores from MIL models based on heterogeneous foundation model features or trained with subgroup-specific configurations showed significant performance improvement as shown in Supplementary Fig. S2.

Contextualizing *EGFR* mutation prediction results for human interpretation is important for conceptual validation (Fig. 1B). Although the interpretation of attention maps has limitations, our results recapitulate the previously described association of histologic features such as lepidic growth, hobnail cytomorphology, and non-solid patterns with *EGFR* mutation (57, 58). Conversely, we demonstrate that specimens with genomically confirmed *EGFR* wild-type status were associated with distinct histologic architecture, such as solid patterns, lymphocytic infiltrate, and clear cell changes, which are again consistent with previous studies. Further analyses using formal evaluations are required to better understand and correlate prediction results with those cardinal histologic features.

In contrast, specimens obtained via FNA exhibit disrupted tissue architecture and reduced tumor cellularity and thus pose a distinct challenge for *EGFR* mutation prediction. However, endobronchial ultrasound-guided FNA is a widely used method for the diagnosis of NSCLC due to its minimally invasive nature and low frequency of procedural-related complications. Therefore, given the wealth of endobronchial ultrasound-guided FNA samples in clinical practice, the application of DL mutation prediction to this specimen class is highly desirable (59, 60). Although only a small proportion of the specimens included in this study were derived by FNA, the ensemble model nevertheless achieved an AUROC of 0.732 for 42 previously unseen FNA specimens across the test sets. This reduced performance compared with that achieved for resection and biopsy specimens may reflect the loss of contextual information from the tumor microenvironment. Albeit these are limited analyses, given the widespread prevalence of cytology samples in the diagnosis of NSCLC, further dedicated studies into this area are warranted in the future. One envisioning of an integrated workflow is for scanning of NSCLC H&E-stained sections immediately after staining, subsequent upload to the cloud for analysis by the DL model, and then a rapid return (e.g., overnight) of prediction results to the responsible pathologist. This capability to provide a prediction with expedited turnaround time (<48 hours) presents an opportunity to rapidly identify patients who are likely to be *EGFR* mutation positive, which can potentially be prioritized for genomic testing (52). Some institutions are already moving toward rapid limited testing (61), and deployment of this DL model will synergize with such developments. Importantly, the use of the computation-only DL model drives more efficient and parsimonious use of “wet” genomic testing and will thus be resource-efficient for oncology care overall.

Going forward, Lunit SCOPE GP can be extended to assess other well-known oncogenic drivers in NSCLC and other tumor types to similarly guide genomic testing and use of matched targeted therapies, extending the principle of rapid resource-efficient testing. Oncogenic alterations in eight genes (*EGFR*, *KRAS*, *ALK*, *MET*, *ROS1*, *BRAF*, *RET*, and *NTRK*) now have FDA- and European Medicines Agency–approved targeted therapies in NSCLC (8, 62). However, given the significantly lesser frequency of these other oncogenic drivers (e.g., 3%–5% for ALK fusions in NSCLC; ref. 63), different strategies for training and model architecture may be needed to engineer robust model performance.

In conclusion, we report the development of a DL model for *EGFR* mutation prediction from routine H&E-stained pathology slides. Through the curation of intentionally diverse training and test datasets, we demonstrate robust performance across key clinical variables previously shown to limit the reach of predictive DL models. This represents a vital step toward translating DL-based genomic prediction into routine clinical workflows. The integration of rapid computational pathology tools, such as that presented here, has the potential to improve outcomes for patients with cancer.

## Supplementary Material

Supplementary Figure S1 Figure S1. Distribution of prediction scores from the ensemble model in Test Set A (n = 1,461) and Test Set B (n = 599).

Supplementary Figure S2 Figure S2. Receiver operating characteristic (ROC) curves for the major three submodels and the ensemble model.

Supplementary Figure S3 Figure S3. Prediction scores of the ensemble model for EGFR mutation prediction across EGFR mutation subtypes in test set A.

Supplementary Figure S4 Figure S4. Receiver operating characteristic (ROC) curves for the data source subgroups (Korea, Republic of Korea; TCGA, The Cancer Genome Atlas Program; US, United States)

Supplementary Figure S5 Figure S5. Prediction scores for EGFR mutation prediction across scanner types in test set C (n = 2,261).

Supplementary Figure S6 Figure S6. Prediction scores for EGFR mutation prediction between two magnifications in test set C (n = 2,261).

Supplementary Figure S7 Figure S7. Model performance across tumor cell count in the test set A (n = 1,461).

Supplementary Figure S8 Figure S8. Model performance across tumor volume (%) in the test set A (n = 1,461).

Supplementary Materials & Methods Supplementary Materials and Methods

Supplementary Table S1 Table S1. Performance comparison between single–multiple instance learning (MIL) approaches and the ABMIL + Slot-MIL approach. Values are presented as AUROC with corresponding 95% confidence intervals (CIs) in brackets.

## Data Availability

Part of the tuning set analyzed in this study was obtained from the NCI CPTAC (available at https://www.cancerimagingarchive.net/collection/cptac-luad/; ref. 36). Part of the test set analyzed in this study was obtained from TCGA Research Network (available at http://cancergenome.nih.gov/; refs. 43, 44). The other data generated in this study are not publicly available because of confidentiality reasons and agreements with raw data providers but are available upon reasonable request from the corresponding authors.
