# Supplementary material for: Deep Learning Predicts EGFR Mutation Status from Histology Images in Non–Small Cell Lung Cancer
Source: Cancer Res Commun. 2025 Dec 8;5(12):2127–41. doi: 10.1158/2767-9764.CRC-25-0155 (PMC12682618; doi:10.1158/2767-9764.CRC-25-0155)
Supplement: Supplementary Figure S1 — Figure S1. Distribution of prediction scores from the ensemble model in Test Set A (n = 1,461) and Test Set B (n = 599). [file crc-25-0155_supplementary_figure_s1_suppsf1.docx]

**
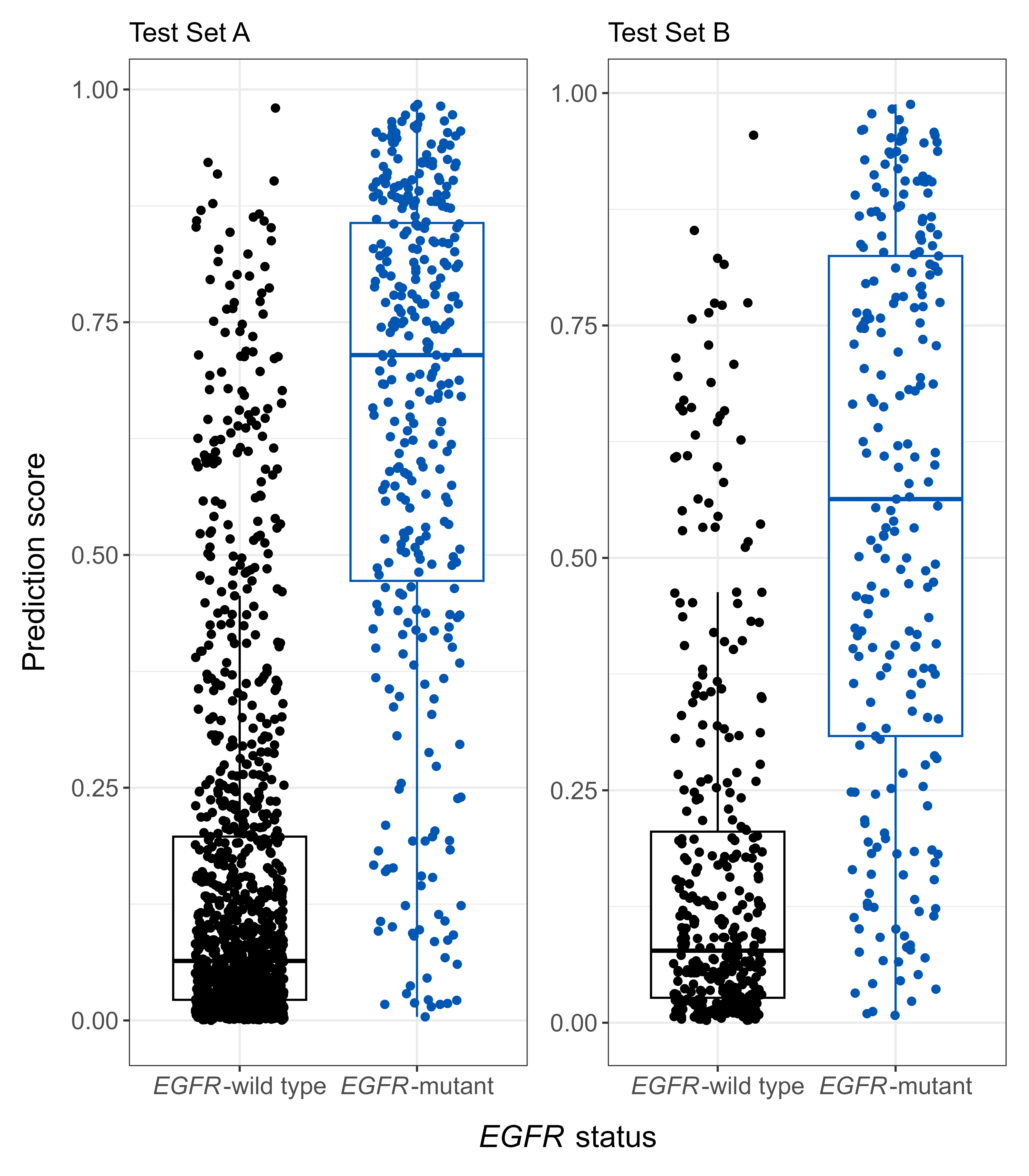
**

**Supplementary Figure S1**. **Distribution of prediction scores from the ensemble model in Test Set A (*n* = 1,461) and Test Set B (*n* = 599).**

*EGFR* mutation prediction scores across test set A and test set B. The data shown represent the median and interquartile range; Test set A, *n* = 1,130 and 331 *EGFR*-wild type and *EGFR*-mutant samples, respectively; Test set B, *n* = 370 and 229 *EGFR*-wild type and *EGFR*-mutant samples, respectively.
