# Supplementary material for: Deep Learning Predicts EGFR Mutation Status from Histology Images in Non–Small Cell Lung Cancer
Source: Cancer Res Commun. 2025 Dec 8;5(12):2127–41. doi: 10.1158/2767-9764.CRC-25-0155 (PMC12682618; doi:10.1158/2767-9764.CRC-25-0155)
Supplement: Supplementary Figure S2 — Figure S2. Receiver operating characteristic (ROC) curves for the major three submodels and the ensemble model. [file crc-25-0155_supplementary_figure_s2_suppsf2.docx]

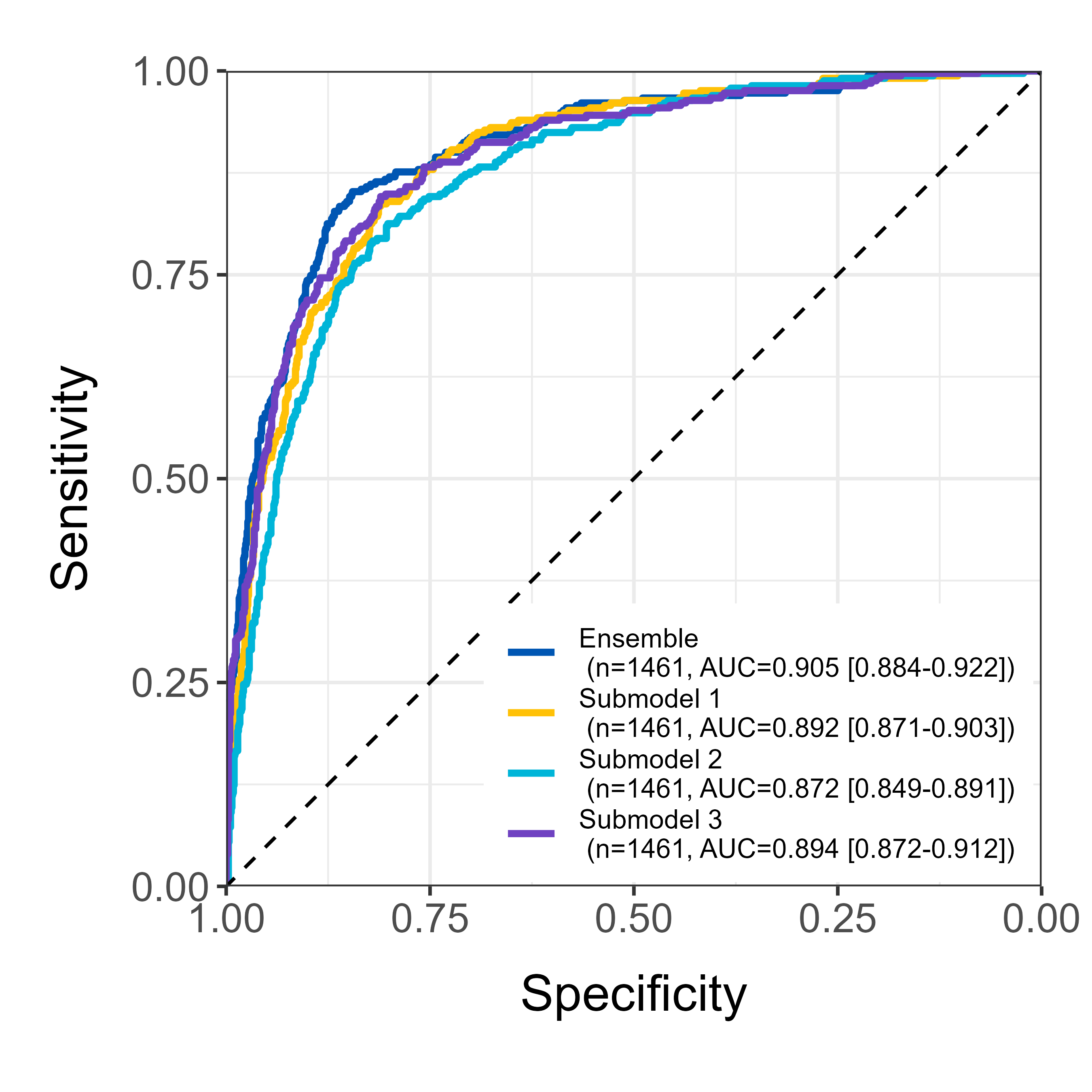


**Supplementary Figure S2. Receiver operating characteristic (ROC) curves for the major three submodels and the ensemble model.**

The submodels were trained with specific subgroups (population, specimen types and histology types) and the ensemble was developed combining predictions from multiple submodels. The 3 submodels shown have the largest ensemble weights, and were trained with the full training population. The figure indicates the area under the ROC curve (AUROC) together with the corresponding 95% confidence intervals.
