# Supplementary material for: Deep Learning Predicts EGFR Mutation Status from Histology Images in Non–Small Cell Lung Cancer
Source: Cancer Res Commun. 2025 Dec 8;5(12):2127–41. doi: 10.1158/2767-9764.CRC-25-0155 (PMC12682618; doi:10.1158/2767-9764.CRC-25-0155)
Supplement: Supplementary Figure S3 — Figure S3. Prediction scores of the ensemble model for EGFR mutation prediction across EGFR mutation subtypes in test set A. [file crc-25-0155_supplementary_figure_s3_suppsf3.docx]

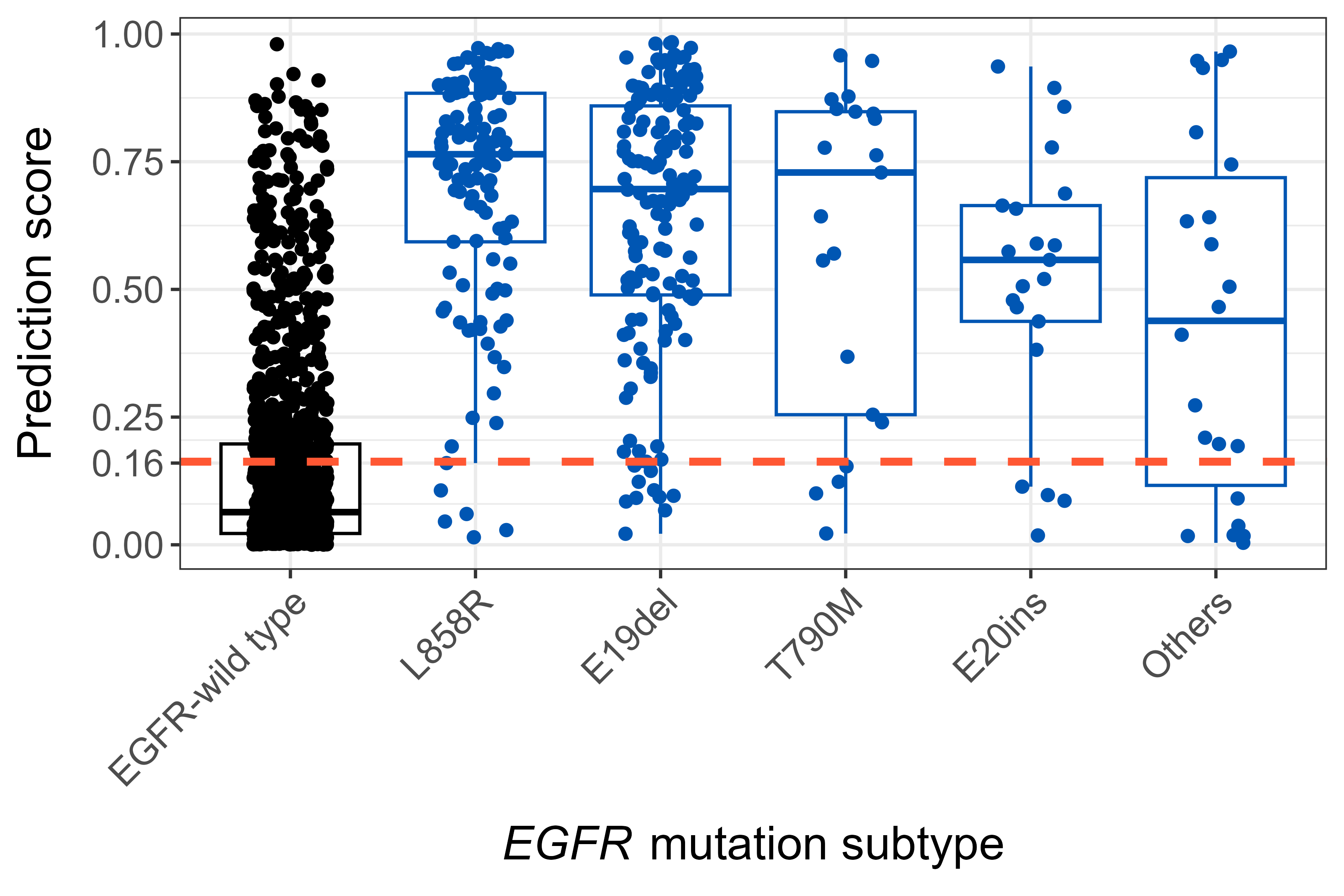


**Supplementary Figure S3. Prediction scores of the ensemble model for *EGFR* mutation prediction across *EGFR* mutation subtypes in test set A.**

*EGFR* mutation subtypes were classified as exon 19 deletions (E19del), L858R point mutation, exon 20 insertions (E20ins), T790M point mutation (including co-mutation with T790M), and uncommon *EGFR* mutations (denoted “Others”). Two samples with E19del+L858R and two samples with L858R+Others were included in the L858R group. The binary cutoff for *EGFR* mutation, determined based on the 90% sensitivity threshold using the tuning set, is shown as a red dashed line. The data shown represent the median and interquartile range; *EGFR*-wild type, *n* = 1130; L858R, *n* = 113; E19del, *n* = 150; T790M, *n* = 21; E20ins, *n* = 21; others, *n* = 22.
