# Supplementary material for: Deep Learning Predicts EGFR Mutation Status from Histology Images in Non–Small Cell Lung Cancer
Source: Cancer Res Commun. 2025 Dec 8;5(12):2127–41. doi: 10.1158/2767-9764.CRC-25-0155 (PMC12682618; doi:10.1158/2767-9764.CRC-25-0155)
Supplement: Supplementary Figure S4 — Figure S4. Receiver operating characteristic (ROC) curves for the data source subgroups (Korea, Republic of Korea; TCGA, The Cancer Genome Atlas Program; US, United States) [file crc-25-0155_supplementary_figure_s4_suppsf4.docx]

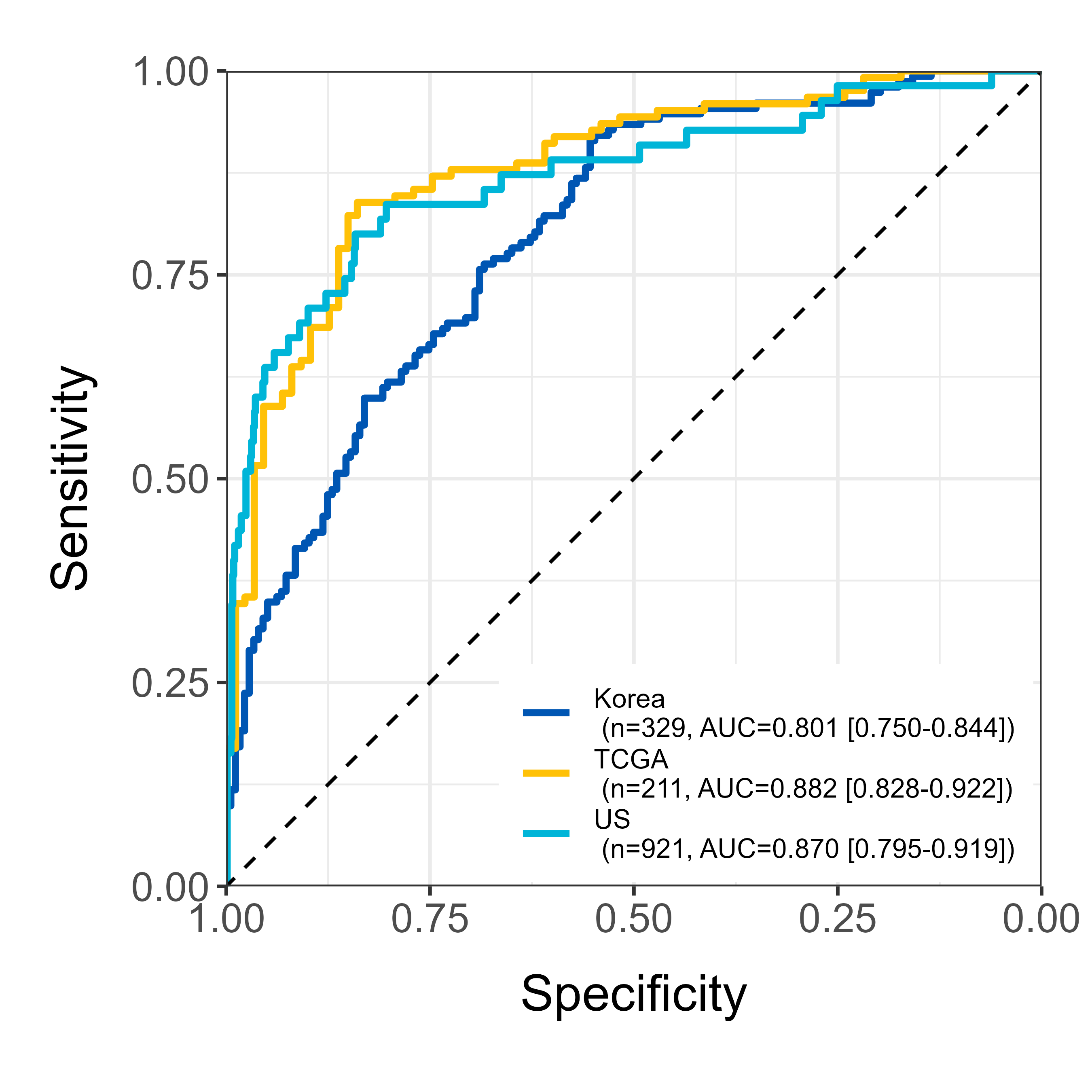


**Supplementary Figure S4**. **Receiver operating characteristic (ROC) curves for the data source subgroups (Korea, Republic of Korea; TCGA, The Cancer Genome Atlas Program; US, United States)**

The figure indicates the area under the ROC curve (AUROC) together with the corresponding 95% confidence intervals.
