# Supplementary material for: Deep Learning Predicts EGFR Mutation Status from Histology Images in Non–Small Cell Lung Cancer
Source: Cancer Res Commun. 2025 Dec 8;5(12):2127–41. doi: 10.1158/2767-9764.CRC-25-0155 (PMC12682618; doi:10.1158/2767-9764.CRC-25-0155)
Supplement: Supplementary Figure S6 — Figure S6. Prediction scores for EGFR mutation prediction between two magnifications in test set C (n = 2,261). [file crc-25-0155_supplementary_figure_s6_suppsf6.docx]

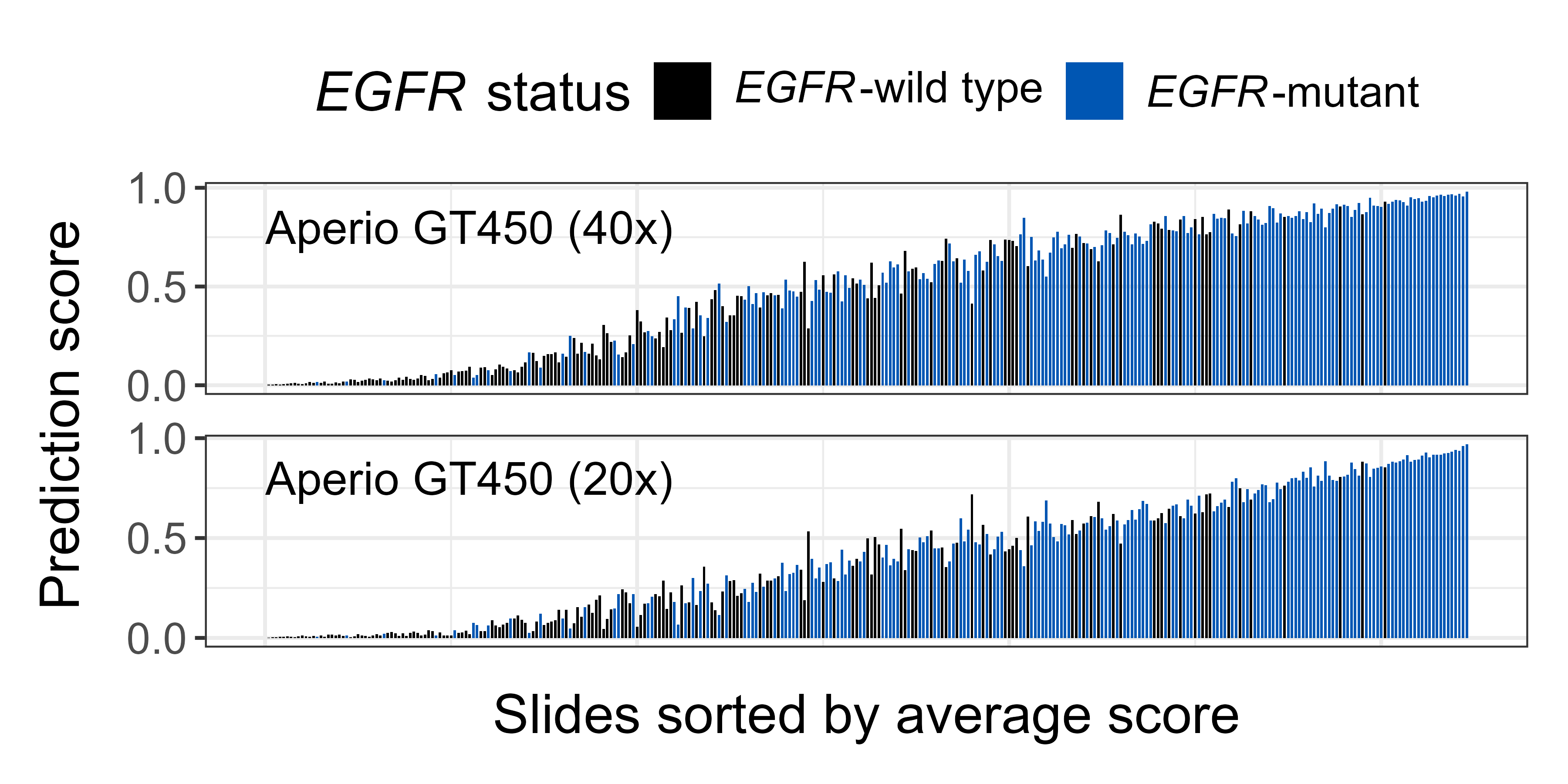


**Supplementary Figure S6. Prediction scores for *EGFR* mutation prediction between two magnifications in test set C (*n* = 2,261).**

The x-axis represents the slides sorted by average value of prediction scores from two magnifications. *EGFR* status based on PCR or NGS was denoted as color (blue, *EGFR*-mutant; black, *EGFR* wild-type).
