# Supplementary material for: Deep Learning Predicts EGFR Mutation Status from Histology Images in Non–Small Cell Lung Cancer
Source: Cancer Res Commun. 2025 Dec 8;5(12):2127–41. doi: 10.1158/2767-9764.CRC-25-0155 (PMC12682618; doi:10.1158/2767-9764.CRC-25-0155)
Supplement: Supplementary Figure S7 — Figure S7. Model performance across tumor cell count in the test set A (n = 1,461). [file crc-25-0155_supplementary_figure_s7_suppsf7.docx]

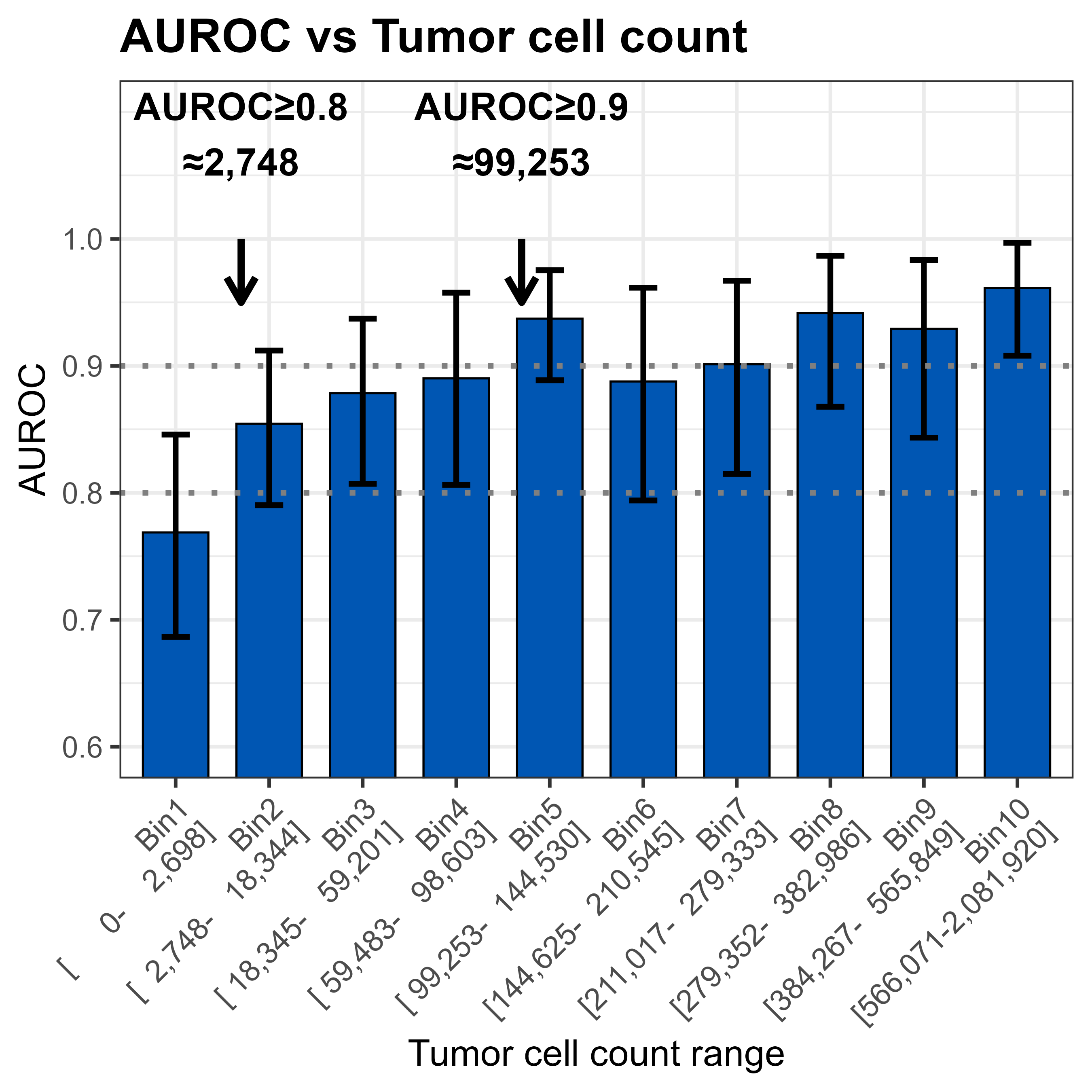


**Supplementary Figure S7. Model performance across tumor cell count in the test set A (*n* = 1,461).**

The distribution of tumor volume was divided into 10 equal-frequency bins (~146 cases per bin) to ensure stable estimation of the area under the receiver operating characteristic curve (AUROC). For each bin, the AUROC is plotted with corresponding 95% confidence intervals. The minimum tumor cell counts required to achieve AUROC thresholds of 0.80 and 0.90 are indicated by the arrows in the figure.
