# Supplementary material for: Deep Learning Predicts EGFR Mutation Status from Histology Images in Non–Small Cell Lung Cancer
Source: Cancer Res Commun. 2025 Dec 8;5(12):2127–41. doi: 10.1158/2767-9764.CRC-25-0155 (PMC12682618; doi:10.1158/2767-9764.CRC-25-0155)
Supplement: Supplementary Materials & Methods — Supplementary Materials and Methods [file crc-25-0155_supplementary_materials__methods_suppsmm.docx]

Supplementary Materials and Methods

MIL model architecture and hyperparameters

We chose ABMIL and Slot-MIL models as they are widely used models with strong performance on our internal test sets. We compared the performance of using only ABMIL-based models, only Slot-MIL-based models and the ensemble of all models considered. In the Tuning set, the combined approach reported an AUROC of 0.858 (95% CI: 0.813, 0.894), which was an improvement over the two baselines (ABMIL: 0.842; Slot-MIL: 0.822). The performance boosts can be observed in Test set A (Combined: 0.905; ABMIL: 0.894; Slot-MIL: 0.898) and Test set B (Combined: 0.860; ABMIL: 0.824; Slot-MIL: 0.850) (**Supplementary Table S1)**. Based on our empirical observation, our hypothesis is that models with different architectures learn to focus on different features of *EGFR*-mutated WSIs due to the inductive biases from the architecture designs. Therefore, based on the performance benefits we adopted the combined MIL approach as our final method.

For the ABMIL model, we use a 3-layered classifier with a gated attention module in the penultimate layer. The hidden dimension size is 512. For the Slot-MIL model, we aggregate patches into four learnable prototype slots, which are then re-aggregated into a single class slot. Slots are initialized using the Xavier uniform distribution with a dimension size of 768. We use 4-head attention, normalizing the attention scores across slots, ensuring the scores between patches and each slot sum to 1. This creates competition among patches for slot attention. During model training, the batch size is chosen as one of [64, 128], and the training step is chosen as one of [2,048, 4,096, 8,192]. Early stopping was applied for efficient training. We used the stochastic gradient descent (SGD) optimizer with an initial learning rate in the range of [1e-2, 1e-4] under the cosine annealing learning rate scheduling. For the training objective function, we selected the binary cross entropy loss with positive weights ranging between 3 and 5. The optimal hyperparameters were chosen by a grid search over all possible configurations.

Ensemble strategy configurations

There are 3 feature types available from the 3 foundation models (ONSSL-FM-CXT, ONSSL-FM-ViT, ST-FM-CXT); 2 classifier architectures available (ABMIL, Slot-MIL); 5 subgroup-specific configurations to train on (population, surgical resection, biopsy, LUAD, non-LUAD). We choose the top 3 models per each subgroup-specific configuration with respect to the tuning set AUROC performance metric. The ensemble score is a weighted average across the chosen models, and the ensemble weights were optimized using the tuning set. The optimization was done by a grid search with 0.05 intervals, while implementing a maximum weight constraint of 0.4 for any individual model to prevent overfitting and avoid undue influence from specific models. These weights underwent optimization using the tuning set and subsequent validation in the test set. The application of subgroup-specific models is precisely targeted to their corresponding sample types. For instance, the resection-specific model's predictions are exclusively utilized for resection samples. For non-LUAD specimens, we employed a modified approach due to the limited quantity of non-LUAD samples in the tuning set, applying a fixed weight of 0.4 to the non-LUAD-specific model for these cases exclusively. As a result, the ensemble model achieved an AUROC of 0.858 on the tuning set.
