# Supplementary material for: Deep Learning Predicts EGFR Mutation Status from Histology Images in Non–Small Cell Lung Cancer
Source: Cancer Res Commun. 2025 Dec 8;5(12):2127–41. doi: 10.1158/2767-9764.CRC-25-0155 (PMC12682618; doi:10.1158/2767-9764.CRC-25-0155)
Supplement: Supplementary Table S1 — Table S1. Performance comparison between single–multiple instance learning (MIL) approaches and the ABMIL + Slot-MIL approach. Values are presented as AUROC with corresponding 95% confidence intervals (CIs) in brackets. [file crc-25-0155_supplementary_table_s1_suppst1.docx]

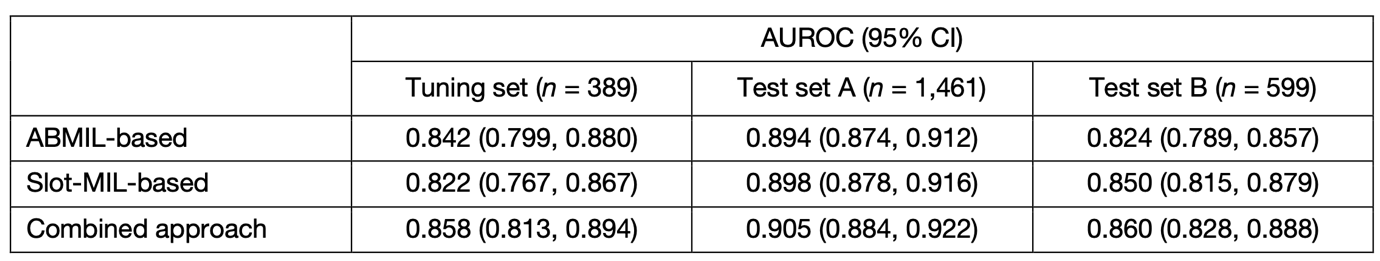


**Supplementary Table S1.** Performance comparison between single–multiple instance learning (MIL) approaches and the ABMIL + Slot-MIL approach. Values are presented as AUROC with corresponding 95% confidence intervals (CIs) in brackets.
